# An Integrated Analog Front‐End System on Flexible Substrate for the Acquisition of Bio‐Potential Signals

**DOI:** 10.1002/advs.202207683

**Published:** 2023-03-03

**Authors:** Runxiao Shi, Xuchi Liu, Tengteng Lei, Lei Lu, Zhihe Xia, Man Wong

**Affiliations:** ^1^ State Key Laboratory of Advanced Displays and Optoelectronics and Technologies Department of Electronic and Computer Engineering The Hong Kong University of Science and Technology Hong Kong 999077 China; ^2^ School of Electronic and Computer Engineering Peking University Shenzhen 518055 China

**Keywords:** analog‐front end systems, flexible electronics, metal‐oxide semiconductors, thin film transistors

## Abstract

The application of a versatile, low‐temperature thin‐film transistor (TFT) technology is presently described as the implementation on a flexible substrate of an analog front‐end (AFE) system for the acquisition of bio‐potential signals. The technology is based on semiconducting amorphous indium‐gallium‐zinc oxide (IGZO). The AFE system consists of three monolithically integrated constituent components: a bias‐filter circuit with a bio‐compatible low cut‐off frequency of ≈1 Hz, a 4‐stage differential amplifier offering a large gain‐bandwidth product of ≈955 kHz, and an additional notch filter exhibiting over 30 dB suppression of the power‐line noise. Respectively built using conductive IGZO electrodes with thermally induced donor agents and enhancement‐mode fluorinated IGZO TFTs with exceptionally low leakage current, both capacitors and resistors with significantly reduced footprints are realized. Defined as the ratio of the gain‐bandwidth product of an AFE system to its area, a record‐setting figure‐of‐merit of ≈86 kHz mm^−2^ is achieved. This is about an order of magnitude larger than the < 10 kHz mm^−2^ of the nearest benchmark. Requiring no supplementary off‐substrate signal‐conditioning components and occupying an area of ≈11 mm^2^, the stand‐alone AFE system is successfully applied to both electromyography and electrocardiography (ECG).

## Graphical abstract

Presently described is The application of a versatile, low‐temperature thin‐film transistor technology to the implementation of a flexible substrate of an analog front‐end system for the acquisition of bio‐potential signals is presen. The analog front‐end system consists of three monolithically integrated constituent components: an area‐saving bias‐filter circuit, a high‐gain 4‐stage differential amplifier, and a notch filter to remove power line noise.

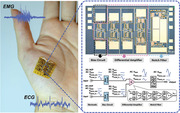

## 1 Introduction

Generated by the trans‐membrane flow of ions,^[^ ^1^, ^2^ ^]^ bio‐potential signals making up electromyogram (EMG),^[^ ^3^ ^]^ electrocardiogram (ECG),^[^ ^4^ ^]^ electrooculogram^[^ ^5^ ^]^ and electroencephalogram^[^ ^6^ ^]^ are acquired for health monitoring^[^ ^7^, ^8^ ^]^ disease diagnosis^[^ ^9^, ^10^ ^]^ and human‐machine interfaces,^[^ ^11^, ^12^ ^]^ etc. Due to the acquisition of low‐operating‐frequency and small‐amplitude bio‐potential signals typically in electrically noisy environments, analog front‐end (AFE) systems providing amplification and filtering are demanded as interfaces between the sensing electrodes and the terminal signal‐processing and ‐rendering systems.^[^ ^13^, ^14^ ^]^ For the comfort of use during signal acquisition, systems realized on flexible substrates are desired.^[^ ^15^, ^16^, ^17^ ^]^ Two important attributes of an AFE system are its gain‐bandwidth product (*GBWP*) and its areal footprint (*S*). A figure‐of‐merit *η* ≡ *GBWP*/*S* can be defined for an AFE system, with a larger *η* corresponding to a more desirable system.

Large *S* is one of the reasons that hinder the improvement of *η*. An AFE system typically includes a high‐pass filter to remove the superfluous DC half‐cell potential^[^ ^18^, ^19^ ^]^ (*V*_HCP_: Table S1, Supporting Information) spontaneously generated at the contact between a sensing electrode and the skin/tissue. With the lowest bio‐potential frequency *f*_C_ of interest (Table S2, Supporting Information) set to ≈1 Hz, a time constant of *τ* > 1 /(2*πf*_C_) ≈ 0.16 s is desired for the high‐pass filter. For a passive “resistor (R)‐capacitor (C)” filter with *τ* ≈ 0.16 s, the components offering the required large capacitance (say *C* ≈ 160 nF) and resistance (say *R* ≈ 1  MΩ) would occupy a large *S*. Furthermore, relatively large R's and C's are also needed in a notch filter for suppressing the 50‐ or 60‐Hz power‐line noise.^[^ ^20^, ^21^ ^]^ Hindered by the technological challenge in implementing large *R* and *C* using monolithically integrated R and C occupying relatively small footprints on a flexible substrate, these components are often packaged off‐substrate.^[^ ^18^, ^22^, ^23^ ^]^

Amplification of a millivolt‐scale bio‐potential to a volt‐scale working potential requires an amplifier with a gain of ≈60 dB. For suppression of the common‐mode noise and to achieve a high gain, a differential amplifier is preferred over a single‐ended amplifier. Compared to an amplifier implemented on silicon supporting a complementary p‐channel load transistor, the relatively lower impedance of the load transistor supported by a unipolar^[^ ^24^, ^25^ ^]^ metal‐oxide thin‐film transistor (TFT) technology results in a smaller differential gain (*A*_d_). Capacitor‐boosting^[^ ^19^, ^26^ ^]^ is accomplished by maintaining a relatively constant potential difference across the gate and source terminals of the load TFT, thus eliminating the shunting transconductance and increasing *A*_d_. Furthermore, the relatively poorer uniformity of TFT device parameters leads to circuit asymmetry, resulting in a finite output offset voltage and the incomplete suppression of common‐mode noise. In an amplifier with large *A*_d_, amplification of a non‐negligible input offset voltage may lead to saturation of its output. A post‐fabrication compensation (PFC)^[^ ^19^, ^26^ ^]^ has been proposed to circumvent the mismatch of TFT parameters.

A high‐level perspective balancing various conflicting demands needs to be adopted when realizing a TFT‐based AFE system on a flexible substrate. The deployment of off‐substrate components to reduce *S* conflicts with the desired malleability attribute of flexible electronics. The addition of large C's to realize capacitor‐boosting increases |*A*_d_| at the expense of bandwidth and *S*. It is unlikely PFC is a practical option since it requires a wasteful provision and tedious characterization of additional TFTs, and manual post‐fabrication configuration of a circuit using printed interconnects. Due to these technological and design constraints, the reported *η* of the state‐of‐the‐art AFE systems on flexible substrates has not exceeded 10 kHz mm^−2^ (Table S3, Supporting Information).

Applying a more versatile low‐temperature TFT technology^[^ ^27^ ^]^ based on semiconducting amorphous IGZO^[^ ^28^, ^29^ ^]^ and incorporating enhanced functionality, the design, and implementation of a stand‐alone AFE system on the flexible substrate are presently described. The technology offers unique features of enhancement‐mode IGZO: F TFTs and conductive IGZO: F electrodes populated with thermally induced donor defects. The exceptionally low leakage current of the former makes it possible to realize small‐footprint R's with suitably high resistance values, and the latter is applied to construct C's with relatively higher capacitance per unit area. These are combined to achieve a significant reduction in *S* while maintaining *f*_C_ < 1 Hz for a high‐pass filter implemented in the form of a bias‐filter circuit. An alternating‐current (AC) coupled with a 4‐stage differential amplifier with extra bias‐filter structures to reduce sensitivity to TFT parameter mismatch is proposed and implemented. As a further enhancement, a notch filter exhibiting over 30 dB suppression of power‐line noise is monolithically integrated. Despite the added functionality, a significantly larger *η* ≈ 86 kHz mm^−2^ is achieved for the presently implemented AFE system. The utility of the system is demonstrated by the acquisition of EMG and ECG signals.

## 2 Results

### 2.1 Monolithic Fabrication of Device Components: TFTs and Capacitors

**Figure 1:**
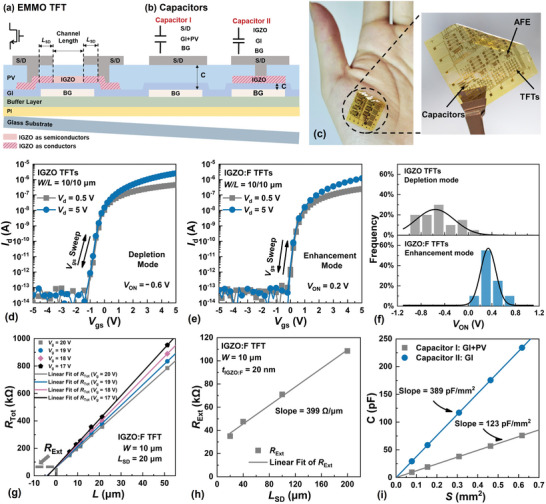
Technology and characteristics of TFTs and capacitors on PI. a,b) Schematic cross‐sections of the EMMO TFT and two different capacitor implementations. c) PI carrying TFTs, capacitors, and AFE systems placed on the thenar muscle of a hand. d,e) Comparison of the transfer characteristics of the depletion‐mode IGZO and enhancement‐mode IGZO: F TFTs. f) Comparison of the *V*_ON_ statistics of 20 IGZO and 20 IGZO:F TFTs. g) *R*_Tot_ of an IGZO: F TFT with *L*_SD_ = 20 µm and *W* = 10 µm at different *V*_g_. h) The dependence of *R*_Ext_ on *L*_SD_ of IGZO:F TFTs with *W* = 10 µm. i) The dependence of the capacitance on the area of Capacitors I and II.

The schematic cross‐sections of an elevated‐metal metal‐oxide (EMMO) IGZO TFT^[^ ^30^, ^31^ ^]^ and C's fabricated on flexible polyimide (PI) substrate are exhibited in **Figure** **1**a,b. For the EMMO TFT with thermally induced source/drain (S/D) regions, the channel length (*L*) is determined by the separation between the S/D electrodes. Monolithically integrated TFTs, C's, and AFE systems on a 20‐µm thick PI placed on the thenar muscle of a hand are shown in Figure 1c.

Fluorine (F) is incorporated in IGZO, thus forming IGZO: F,^[^ ^32^, ^33^ ^]^ by exposure of the IGZO to a tetrafluoromethane (CF_4_) plasma. The transfer characteristics, that is the dependence on the drain current (*I*_d_) on the gate‐to‐source voltage (*V*_gs_), of IGZO and IGZO:F TFTs are displayed respectively in Figure 1d,e. The drain‐to‐source voltage (*V*_ds_) is fixed at 0.5 and 5 V, and the channel width (*W*)/*L* of the TFTs is 10/10 µm. The turn‐on voltage (*V*_ON_) of a TFT is defined as the *V*_gs_ needed to induce a *W*‐normalized *I*_d_ of 1 pA µm^−1^. The statistical distribution of *V*_ON_ over a collection of IGZO:F and IGZO TFTs are compared in Figure 1f. Consistent with the suppressed generation of donor‐defects in IGZO: F,^[^ ^33^, ^34^ ^]^ it is clear that IGZO: F TFTs with *V*_ON_ > 0 V and IGZO TFTs with *V*_ON_ < 0 V exhibit respectively enhancement‐ and depletion‐mode behavior. An IGZO:F TFT biased at *V*_gs_ = 0 V would operate in the off‐state and exhibit a significantly larger channel resistance than that of an IGZO TFT biased at the same *V*_gs_. Consequently, a beneficial application of IGZO:F TFT is the realization of a small‐footprint R with a large resistance. The output characteristics, that is the dependence of *I*_d_ on *V*_ds_, of IGZO and IGZO:F TFTs are also shown in Figure S1 (Supporting Information).

The total resistance (*R*_Tot_) of a collection of IGZO: F TFTs with different *L* but fixed lengths of the S/D regions (*L*_SD_) is measured at different *V*_gs_ and plotted against *L* in Figure 1g. An external resistance (*R*_Ext_) that includes all the resistance components not modulated by *V*_gs_ is obtained from the common intercept of the fitting lines.^[^ ^35^ ^]^ These experiments are repeated for TFTs with different values of *L*_SD_. The dependence of *R*_Ext_ on *L*_SD_ is plotted in Figure 1h and the slope of the fitting line is used to extract a relatively low resistivity of 8 mΩ cm associated with the thermally induced IGZO: F conductor.^[^ ^30^ ^]^ The same method has been applied to IGZO TFTs to extract the resistivity of their S/D regions as shown in Figure S2 (Supporting Information). The similarity between the resistivity values is a good indication that fluorination does not affect the realization of thermally induced IGZO: F conductors. The evolution of the transfer characteristics of IGZO: F TFTs under mechanical and electrical stress is shown in Figure S3 (Supporting Information).

Unlike the C (Figure 1b: Capacitor I) realized using a conventional metal‐oxide TFT technology and consisting of a stack of the passivation layer (PV) on a gate insulator (GI) sandwiched between an S/D electrode and a bottom gate (BG) electrode, an improved C (Figure 1b: Capacitor II) with a larger capacitance per unit area is realized and consisting of only the GI sandwiched between a thermally induced IGZO: F conductor and the BG electrode. In the present implementation, the oxide‐equivalent thickness of the GI is ≈100 nm and that of the PV is ≈220 nm. The dependence of the capacitance on the area of the C's is compared in Figure 1i. Deduced from the slopes of the linear regression fits and consistent with the oxide‐equivalent thickness of the dielectric layers, the capacitance per unit area of Capacitor II is ≈3.2 times larger than that of Capacitor I. When implementing a given capacitance, the footprint of Capacitor II would be ≈30% of that of Capacitor I – leading to a significant reduction in area. The capacitance‐voltage characteristics of Capacitors I and II with the same area of ≈0.31 mm^2^ are shown in Figure S4 (Supporting Information).

### 2.2 Evolution of the Signal Components through the AFE System

**Figure 2:**
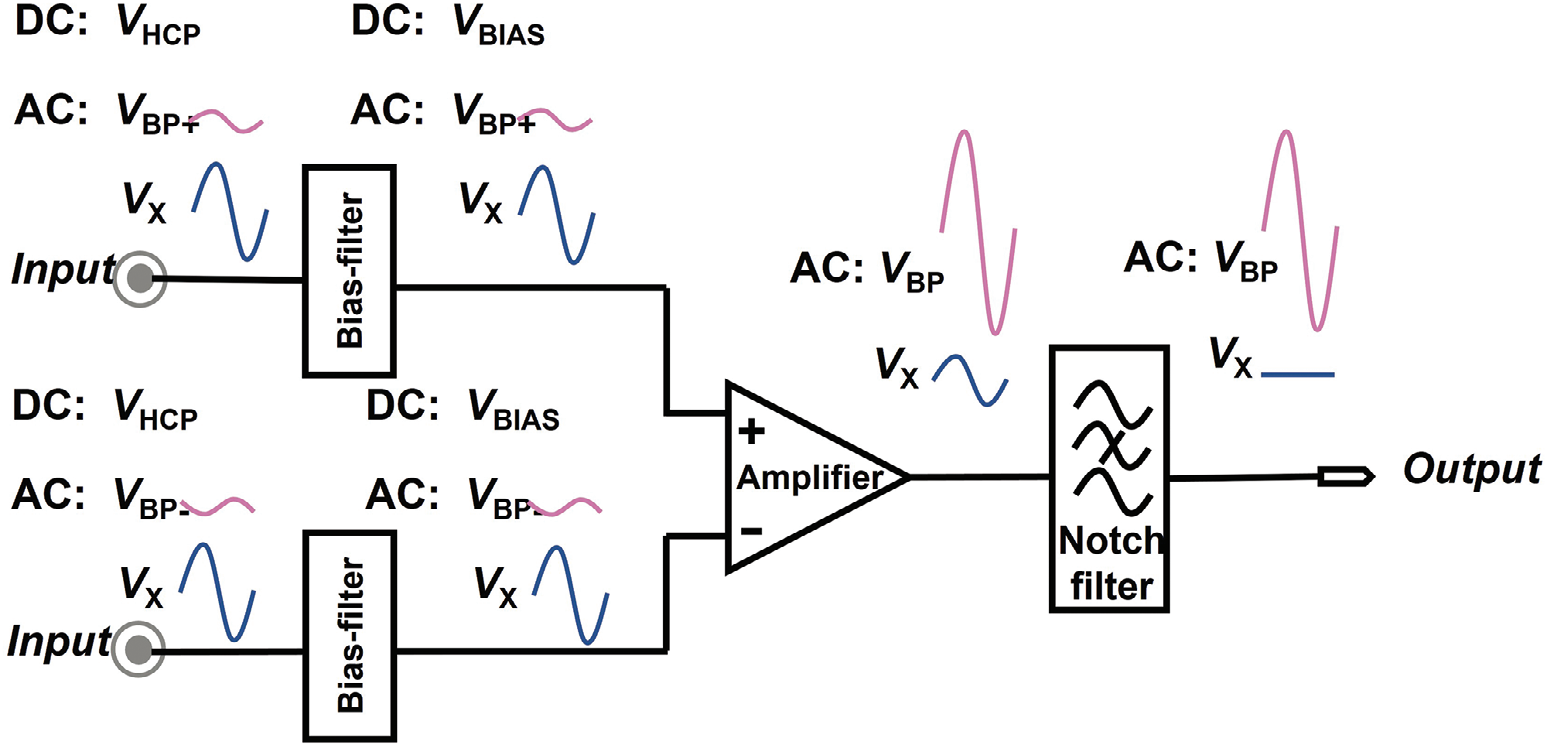
The evolution of the signal components through the constituent components of the AFE system.

The overall architecture of the presently implemented AFE system is shown in **Figure** **2**, consisting of monolithically integrated bias‐filter circuits, differential amplifier, and notch filter. A bias‐filter circuit takes in a signal composed of a DC component of the *V*_HCP_ originating at the sensing electrode and an AC component of a time‐varying bio‐potential (*V*_BP_) with amplitude typically on the order of millivolts. The input signal also includes another time‐varying noise potential *V*_X_, such as the 50‐ or 60‐Hz power‐line noise ($V_{X\_\mathrm{PL}}$) and noise from other untargeted sources. Fed into the next differential amplifier stage is the output of the two bias‐filter circuits, composed of the AC components *V*_BP_, *V*_X_ and a DC component *V*_BIAS_. *V*_BIAS_ is set to match the desired operating point of the amplifier. The amplifier enables the amplification of the differential‐mode *V*_BP_ and the suppression of any common‐mode components of *V*_X_ and *V*_BP_. Fed into the next notch filter stage is the output of the amplifier, composed of the amplified differential‐mode *V*_BP_ and any residual *V*_X_. With the $V_{X\_\mathrm{PL}}$ suppressed by the notch filter and consisting mainly of the amplified *V*_BP_, the output of the notch filter is that of the AFE system.

## 3 2T1C Bias‐Filter Circuit

**Figure 3:**
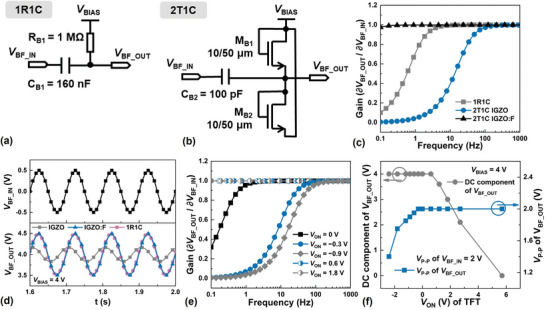
Circuit diagrams and characteristics of the proposed 2T1C bias‐filter circuit. a) Circuit diagram of the traditional 1R1C design and b) that of the proposed 2T1C design. Comparison of c) the frequency response characteristics and d) the sinusoidal response characteristics of the bias‐filters constructed of 1R1C, 2T1C with IGZO TFTs, and 2T1C with IGZO: F TFTs. e,f)The effects of *V*_ON_ shift on the characteristics of the proposed 2T1C bias‐filter circuit.

The DC component of the signal at the input of the present AFE system does not carry bio‐potential information of relevance. An example would be the superfluous *V*_HCP_ at the signal‐acquisition electrode.^[^ ^18^, ^19^ ^]^ A bias‐filter circuit is desired, capable of removing such non‐essential DC signal at its input while superimposing a suitable DC *V*_BIAS_ on the time‐varying *V*_BP_ at its output. An example of a suitable *V*_BIAS_ is one matching the optimal operating point of a differential amplifier. The schematics of bias‐filter circuits constructed of a conventional 1R1C and the presently proposed 2T1C (i.e. 2‐TFTs and 1‐C) high‐pass filters are shown respectively in **Figure** **3**a,b. In the latter, two diode‐connected TFTs are deployed to allow both the charging and discharging of the capacitor. The necessity of deploying an additional diode‐connected discharging TFT is elaborated in Figure S5 (Supporting Information). In the absence of a discharging path, the output voltage $V_{\mathrm{BF}\_\mathrm{OUT}}$ would exhibit a deviation of its DC component from *V*_BIAS_ and a dependence of its peak‐to‐peak voltage swing *V*_P − P_ on the input $V_{\mathrm{BF}\_\mathrm{IN}}$ to the bias circuit. Compared in Figure 3c are the simulated frequency response characteristics of i) the 1R1C design with resistance *R* = 1 MΩ and capacitance *C* = 160 nF, ii) a 2T1C design deploying enhancement‐mode IGZO:F TFTs with *W*/*L* = 10/50  µm and a more than 3 orders‐of‐magnitude smaller *C* = 100 pF, and iii) a 2T1C design deploying depletion‐mode IGZO TFTs with the same *W*/*L* and *C* = 100 pF. The respective *f*_c_’s are ≈1, < 0.1 and ≈20 Hz. These results are expected, since an enhancement‐ or a depletion‐mode diode‐connected TFT with 0 V applied across its terminals would operate respectively in a highly resistive “off” state or a relatively more conductive “on” state.

The corresponding sinusoidal response characteristics are compared in Figure 3d. The behavior of the bias‐filter circuits constructed of 2T1C with enhancement‐mode IGZO: F TFTs and 1R1C are similar, imparting a new *V*_BIAS_ = 4 V at their $V_{\mathrm{BF}\_\mathrm{OUT}}$ while retaining the *V*_P − P_ of 1 V of a 10‐Hz sinusoidal $V_{\mathrm{BF}\_\mathrm{IN}}$ with an initial DC bias of 0 V. However, for the bias‐filter circuit constructed of 2T1C with the less resistive depletion‐mode IGZO TFTs, the resulting *V*_P − P_ ≈ 0.5 V of the corresponding $V_{\mathrm{BF}\_\mathrm{OUT}}$ is significantly attenuated.

The different behavior of the two 2T1C designs is further illustrated with the frequency response characteristics shown in Figure 3e. The more positive the *V*_ON_, the higher the channel resistance of the diode‐connected TFT, and the lower the *f*_c_ of the resulting high‐pass filter. The actual resistance of a diode‐connected TFT is correlated with the off‐state leakage current of the TFT. A *W*‐normalized leakage current of less than 10^−18^ A µm^−1^ has been reported,^[^ ^36^ ^]^ an exceptionally low value. The dependence of *V*_P − P_ and *V*_BIAS_ on *V*_ON_ are displayed in Figure 3f, showing more deterioration of the former with more negative *V*_ON_, thus highlighting again the advantage of an enhancement‐mode IGZO:F TFT over a depletion‐mode IGZO TFT. On the other hand, an excessively positive *V*_ON_ should also be avoided since it would lead to an undesirably long time for $V_{\mathrm{BF}\_\mathrm{OUT}}$ to settle at *V*_BIAS_. A comparison of the layouts of the bias‐filter circuits constructed of 1R1C and 2T1C is shown in Figure S6 (Supporting Information), with the latter occupying a footprint merely ≈1/1600 times of the former.

## 4 4‐Stage AC‐Coupled Differential Amplifier

**Figure 4:**
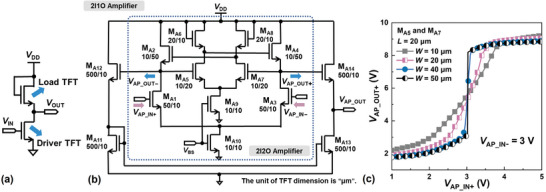
4‐stage AC‐coupled differential amplifier. a) Inverter amplifier consisting of a load‐ and a driver‐TFT. b) Circuit diagram of a 2I1O amplifier containing a core 2I2O amplifier. c) $V_{\mathrm{AP}\_\mathrm{OUT}+}$ versus $V_{\mathrm{AP}\_\mathrm{IN}+}$ voltage‐transfer characteristics at *V*_IN −_ = 3 V for both M_A5_ and M_A7_ with *W*/*L* of 20/10 µm or 50/10 µm. As *A*_f_ is increased with increasing *W* and made to approach 1, |*A*_d_| in the transition region of the transfer, characteristics increase accordingly.

Consisting of a load‐ and a driver‐transistor (**Figure** **4**a), an inverter is the fundamental building block of an amplifier. The *A*_d_ of an inverter amplifier is given by the product of the output resistance (*r*_o_) of the load‐transistor and the transconductance (*g*_m_) of the driver‐transistor.^[^ ^37^ ^]^ Due to the lack of a viable technology for implementing a p‐type metal‐oxide TFT load, a relatively small *r*_o_ limits the gain of an inverter amplifier based on unipolar TFTs. Capacitor‐boosting^[^ ^19^, ^20^ ^]^ has been deployed to increase the *r*_o_ by maintaining a relatively constant potential difference across the gate and source terminals of the load‐TFT. However, this method leads to a reduction in the bandwidth and a significant increase in *S* of the amplifier, as shown in Figure S7 (Supporting Information). The circuit diagram of a differential amplifier potentially offering a higher gain is shown in Figure 4b, with ideally identical driver TFTs M_A1_, M_A3_ and ideally identical load TFTs M_A2_, M_A4_ forming a symmetrical basis pair providing differential amplification.^[^ ^38^ ^]^ An ideally symmetrical differential pair consisting of TFTs M_A5_ ≈ M_A8_ is inserted to provide the gain‐boosting positive feedback. TFTs M_A9_ and M_A10_ are used to implement the current sources to provide the negative feedback needed for suppressing the common‐mode components of the differential inputs $V_{\mathrm{AP}\_\mathrm{IN}+}$ and $V_{\mathrm{AP}\_\mathrm{IN}-}$. This collection of TFTs forms a 2‐input/2‐output (2I2O) differential amplifier, with $V_{\mathrm{AP}\_\mathrm{OUT}+}$ and $V_{\mathrm{AP}\_\mathrm{OUT}-}$ as the differential outputs. TFTs M_A11_ ≈ M_A14_ are added to convert the 2 outputs to 1 output, thus realizing a 2‐input/1‐output (2I1O) differential amplifier.

The *A*_d_ of an ideal 2I2O amplifier is given below:

(1) $$A_{d}=-g_{m1}\left[\frac{1}{g_{m2}\;\left(1-A_{f}\right)}∥r_{o2}∥r_{o1}\right]$$

(2) $$A_{f}=g_{m7}\;\left(\frac{1}{g_{m8}\;}∥r_{o7}∥r_{o8}\right)\approx \frac{g_{m7}}{g_{m8}}$$

where *A*_f_ is the gain‐boosting feedback factor determined by the relative dimensions of TFTs M_A7_ and M_A8_. The numbers *α* and *β* appearing respectively in *g*_m*α*_ and *r*_o*β*_ refer to the corresponding numerical designations of the TFTs in Figure 4b. It can be seen from Equation (1) that |*A*_d_| can be significantly increased when *A*_f_ is made to approach 1. This is illustrated in Figure 4c, showing a series of $V_{\mathrm{AP}\_\mathrm{OUT}+}$ versus $V_{\mathrm{AP}\_\mathrm{IN}+}$ voltage‐transfer characteristics while fixing $V_{\mathrm{AP}\_\mathrm{IN}-}\;=\;\;3$ V. As *A*_f_ is increased and made to approach 1 by increasing the *W* of the driver TFT M_A7_, |*A*_d_| in the transition region of the transfer characteristics increases accordingly.

**Figure 5:**
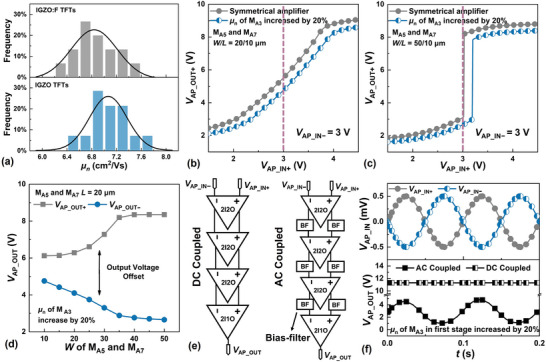
Problems presented by the variation of TFT parameters in the differential amplifier. a) The statistical distributions of µ_n_ of IGZO: F and IGZO TFTs. b,c) Variation of the voltage‐transfer characteristics due to TFT mismatch, the µ_n_ of M_A3_ is 20% higher compared with that of M_A2_. *W*/*L* of both M_A5_ and M_A7_ are 20/10 or 50/10 µm, representing amplifier with respectively low and high |*A*_d_|. d) Output voltage offset due to the variation of TFT parameters. e) Cascades of DC‐ and AC‐coupled amplifiers. f) Comparison of the characteristics of 4‐stage DC‐ and AC‐coupled differential amplifiers in the presence of TFT µ_n_‐mismatch.

It is not always desirable to make |*A*_d_| very large by making *A*_f_ approach 1, particularly in the inevitable presence of TFT parameter variations. Besides the *V*_ON_ variation shown earlier in Figure 1f, exhibited in **Figure** **5**a is the statistical distributions of the field‐effect mobility µ_n_. The respective average and standard deviation for IGZO: F TFTs are 6.8 and 0.7 cm^2^ V^−1^ s^−1^, while the same for IGZO TFTs are 7.1 and 0.6 cm^2^ V^−1^ s^−1^, respectively. The effects of such parameter variation are illustrated in Figure 5b,c, showing the voltage transfer characteristics of differential amplifiers with the µ_n_ of TFT M_A3_ made 20% higher than that of TFT M_A2_, thus breaking the symmetry of an ideal amplifier. For $V_{\mathrm{AP}\_\mathrm{IN}+}$ around $V_{\mathrm{AP}\_\mathrm{IN}-}\;=\;3$ V, the relatively lower peak‐|*A*_d_| of an asymmetrical amplifier with *W*  = 10 µm for TFT M_A7_ is maintained while |*A*_d_| is greatly attenuated for a high‐gain asymmetrical amplifier with *W* = 50 µm for TFT M_A7_. In order to maintain a reasonable |*A*_d_| over a relatively larger input dynamic range, it is desirable to implement a cascaded multi‐stage amplifier, with each stage in the cascade designed to offer a relatively modest |*A*_d_|. Presently, the number of stages is set to 4, with three 2I2O differential amplifiers terminating in one 2I1O differential amplifier.

The variation of TFT parameters presents another problem when cascading amplifiers. This is illustrated in Figure 5d, showing the appearance of an offset between the differential outputs, that is $V_{\mathrm{AP}\_\mathrm{OUT}+}\;\neq \;V_{\mathrm{AP}\_\mathrm{OUT}-}$, even when $V_{\mathrm{AP}\_\mathrm{IN}+}\;=\;V_{\mathrm{AP}\_\mathrm{IN}-}\;$. Such an offset, if excessive and not eliminated, might cause saturation when presented at the input terminals and amplified by the next amplifier stage. Compared with the DC‐coupled amplifier design, an AC‐coupled amplifier with a high‐pass filter at the output of an amplifier in each stage can have this offset filtered out. Clearly, the same 2T1C bias‐filter circuit can be beneficially deployed as a high‐pass filter to realize the AC coupling. Shown in Figure 5e is a schematic of the presently proposed 4‐stage amplifier. The simulation results in Figure 5f demonstrate the need for the proposed AC coupling in the presence of a TFT mismatch.

**Figure 6:**
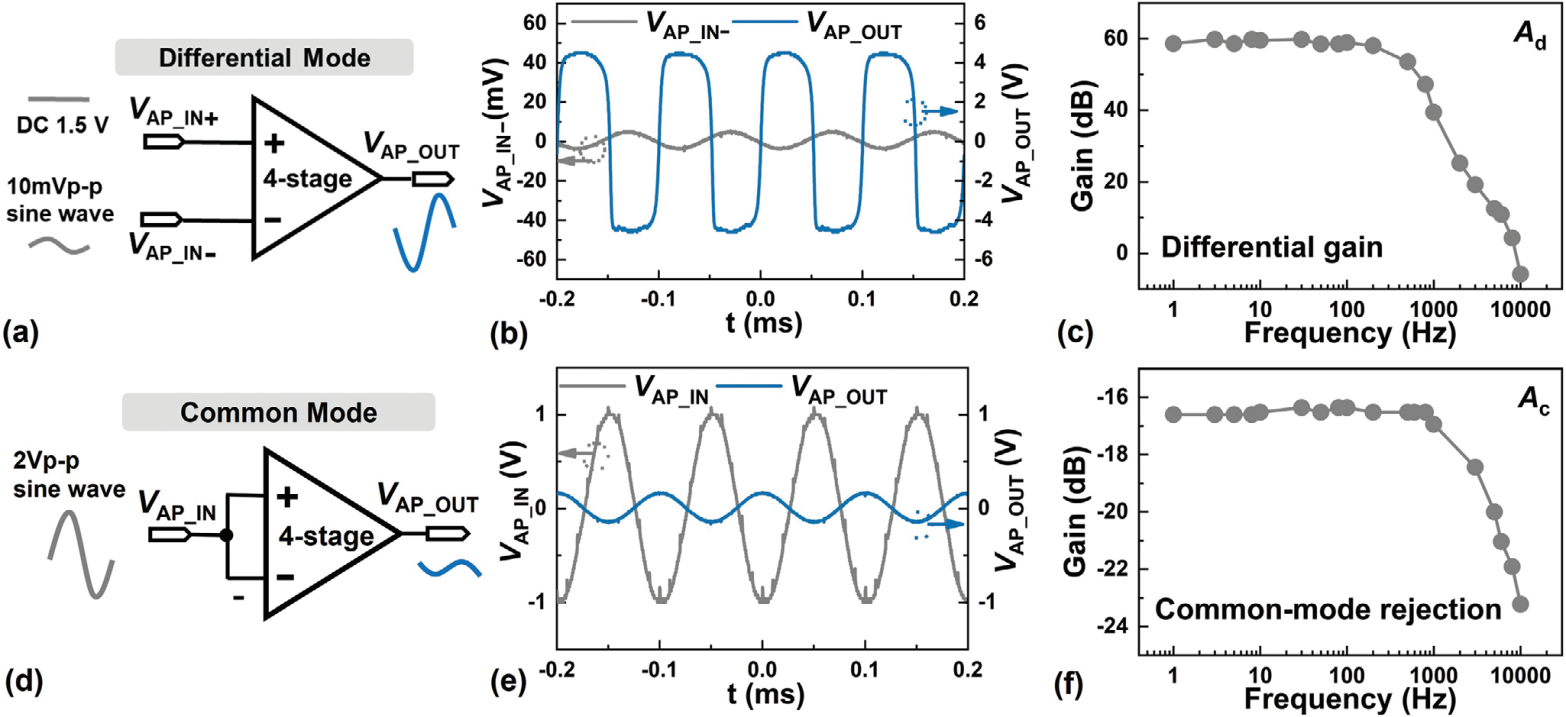
Measurement results of the 4‐stage AC‐coupled differential amplifier. a) Setup for differential‐mode measurement. b) Measured input and output waveforms with 10‐Hz sinusoid differential‐mode input with *V*_P − P_ = 10 mV. c) Frequency response characteristic under differential‐mode measurement. d) Setup for common‐mode measurement. e) Measured input and output waveforms with 10‐Hz sinusoid common‐mode input with *V*_P − P_ = 2 V. f) Frequency response characteristic under common‐mode measurement.

Shown in **Figure** **6** are the measured characteristics of a 4‐stage AC‐coupled amplifier in response to differential‐ and common‐mode input signals. For the differential‐mode measurement shown in Figure 6a, $V_{\mathrm{AP}\_\mathrm{IN}+}\;=\;1.5$ V and $V_{\mathrm{AP}\_\mathrm{IN}-}$ is a 10‐Hz sinusoidal signal with a *V*_P − P_ = 10 mV. It can be seen in Figure 6b that the *V*_P − P_ of the output is ≈9.1  V, reflecting an effective |*A*_d_| ≈ 59.2 dB. The frequency response characteristic is shown in Figure 6c, indicating a bandwidth of ≈1 kHz. This covers nicely the effective frequency range of a variety of bio‐potential signals. A *GBWP* ≈ 955 kHz can be calculated by combining the measured gain and the bandwidth. It should be pointed out that there is a certain amount of distortion of the output waveform shown in Figure 6b. This is because the *V*_P − P_ is distorted by saturation when the output exceeds the maximum output range of the amplifier. If the gain of each individual amplifier in the cascade is measured separately (Figure S8, Supporting Information), one would obtain an aggregated |*A*_d_| ≈ 70 dB. For the common‐mode measurement setup shown in Figure 6d, $V_{\mathrm{AP}\_\mathrm{IN}+}\;=\;V_{\mathrm{AP}\_\mathrm{IN}-}\;$ is a 10‐Hz sinusoidal signal with a *V*_P − P_ = 2 V. It can be seen in Figure 6e that the *V*_P − P_ of the output is ≈0.3 V, reflecting a common‐mode suppression *A*_c_ of ≈16.5 dB. When combined with the effective |*A*_d_| ≈ 59.2 dB, this gives rise to a common‐mode rejection ratio (*CMRR*) of 75.7 dB. The corresponding frequency response characteristic is shown in Figure 6f.

### 4.1 Tunable Notch Filter

**Figure 7:**
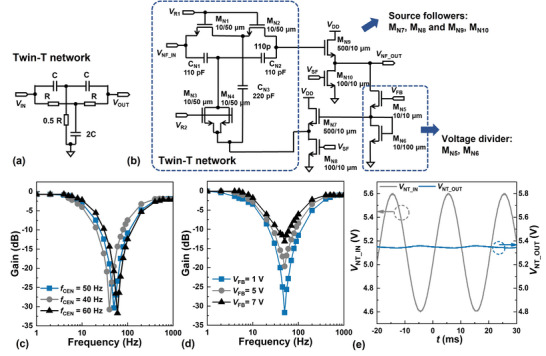
Measurement results of the tunable notch filter. a) Circuit diagram of the R‐ and C‐ based “twin‐T” notch filter. b) Circuit diagram of the proposed “twin‐T” structure based on TFTs. c) Tunning of the *f_CEN_* of the notch filter by changing the gate voltage of M_N1_ ≈ M_N4_. d) Tunning the bandwidth of the notch filter by adjusting the voltage divider consisting of M_N7_ and M_N8_. e) The notch filter showing suppression by −32 dB of a 50‐Hz sinusoidal waveform.

A notch filter is deployed to suppress the power‐line noise. Commonly implemented using an R‐ and C‐based “twin‐T” network (**Figure** **7**a), in which the values of the R's and C's determine a center frequency *f*_CEN_ tuned to the 50‐ or 60‐Hz of the power‐line frequency.^[^ ^39^ ^]^ Since such low *f*_CEN_ demands the deployment of relatively large R's and C's, an off‐substrate filter is often deployed for an AFE system on a flexible substrate.^[^ ^26^, ^40^ ^]^ Because of the relatively low µ_n_ ≈ 6 cm^2^ V^−1^ s^−1^ of IGZO:F, significantly smaller TFTs can be deployed to replace the R's in the “twin‐T” network. Enabled also by the availability of C's with relatively high capacitance per unit area, a first attempt is presently reported on the implementation on a flexible substrate of monolithically integrated “twin‐T” notch filter with the resistors replaced with IGZO: F TFTs (Figure 7b).

The *f*_CEN_ (Figure 7c) and the bandwidth (Figure 7d) of the twin‐T network are regulated respectively by adjusting the bias *V*_R1_ and *V*_R2_ applied on the R‐replacing TFTs M_N1_ ≈ M_N4_ and the feedback bias *V*_FB_ of the voltage divider consisting of TFTs M_N5_ and M_N6_. The percentage of DC components fed back into the “twin T” network from the output affects the bandwidth of the notch filter by modulating the quality factor.^[^ ^41^ ^]^ The source follower consisting of TFTs M_N7_ and M_N8_ is deployed to couple the signal from the voltage divider to the twin‐T network. It can be seen in Figure 7e that the ability to suppress the signal at *f*_CEN_ diminishes with reducing amplitude. With the gate capacitance of TFT M_N9_ made ≈50 times smaller than the capacitance of the C's in the twin‐T network to prevent the shunting of high‐frequency signal through the gate electrode of M_N9_, the source follower consisting of TFTs M_N9_ and M_N10_ is used to isolate the twin‐T network when delivering the output $V_{\mathrm{NF}\_\mathrm{OUT}}$ of the notch filter to its load. For the voltage followers, *V*_SF_ is set to a relatively small value to wider the voltage range over which M_N8_ and M_N10_ operate in the desired saturation mode and M_N7_ and M_N9_ are widened to minimize the difference between the DC components of the input and output signals.

Summaries of the values of the various bias settings used to obtain the measurement results shown in Figures 7c and 6d are given respectively in Tables S4,S5 (Supporting Information). Shown in Figure 7e is the input ( *V*_P − P_ = 1 V) and output ( *V*_P − P_ = 25 mV) voltage waveforms of the notch filter, exhibiting a suppression by ≈32 dB of the 50‐Hz power‐line noise. To the best of the authors’ knowledge, this is the first reported 2T1C tunable notch filter fabricated on a flexible substrate.

### 4.2 In Vitro Measurement of EMG and ECG

**Figure 8:**
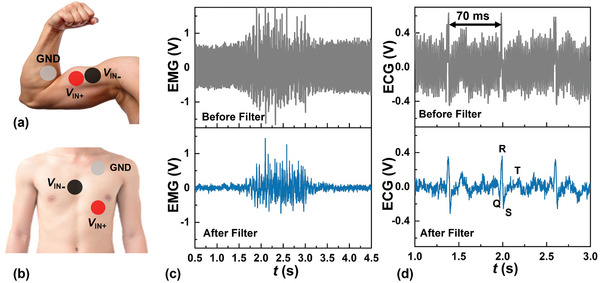
In‐vitro measurement of the EMG and ECG using the fabricated AFE system. Placement of the three electrodes during the acquisition of a) EMG and b) ECG. c) EMG signals acquired when the biceps are exercised. d) ECG signals acquired from the chest. By using the notch filter, the power‐line noise is clearly reduced in both EMG and ECG.

The AFE system has been applied to the in‐vitro measurement of EMG and ECG. Silver/silver chloride electrodes are applied to the surface of the skin and are directly connected to the AFE system. The output of the AFE is directly fed into and rendered on an oscilloscope. A total of three electrodes are deployed, with two connected to the *V*_IN +_, *V*_IN −_ and one connected to the ground of the AFE system. The placement of the electrodes during the acquisition of EMG and ECG is shown respectively in **Figure** **8**a,b respectively.

Shown in Figure 8c is the EMG signal acquired when the biceps are exercised. Shown in Figure 8d is the ECG signal acquired from the chest, exhibiting a heart rate of 86 beats min^−1^. Clearly, the 50‐Hz background power‐line noise in both measurements has been effectively suppressed by the notch filter, with the Q, R, S, and T^[^ ^42^ ^]^ features more readily discerned in the lower figure of Figure 8d.

## 5 Summary

**Figure 9:**
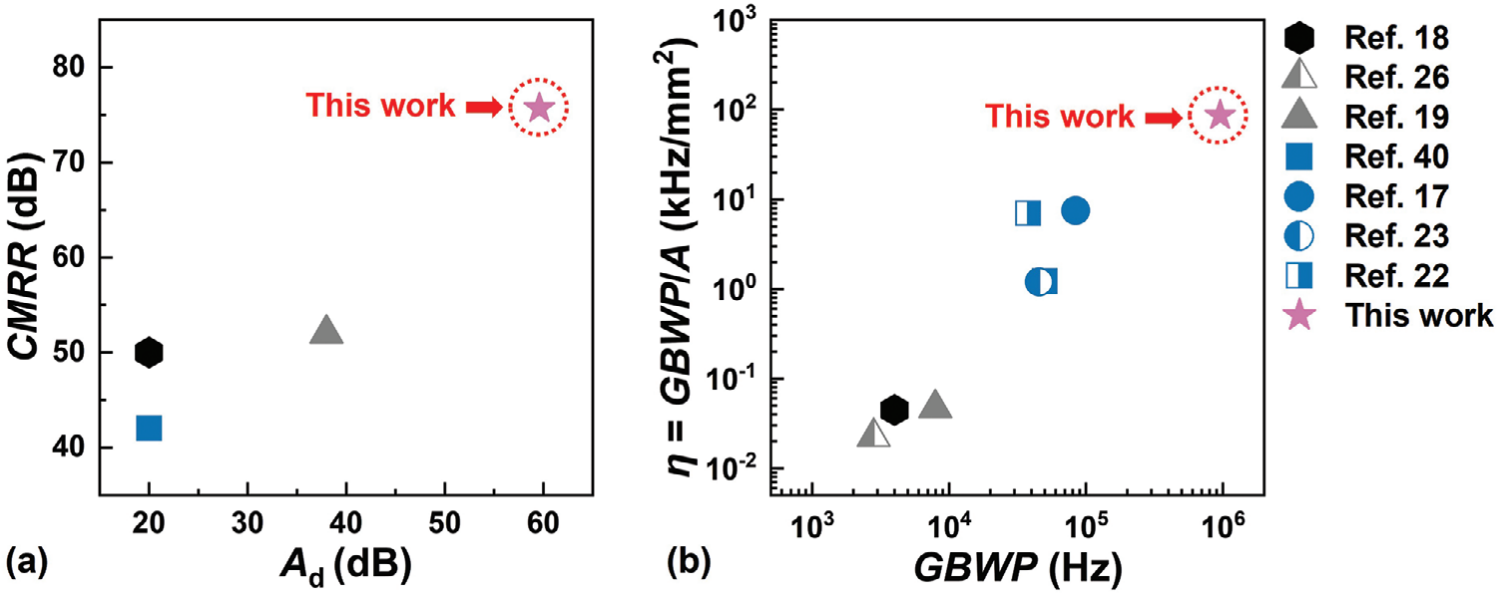
Comparison of the proposed AFE with published state‐of‐the‐art systems. a) Comparison of *A*_d_ and *CMRR*. b) Comparison of *GBWP* and *η*. Deployed respectively in Refs. (18), (26 and 19), and (40, 17, 23, and 22) are amorphous silicon, organic, and metal‐oxide TFTs.

Based on a versatile, low‐temperature, amorphous IGZO TFT technology, an AFE system on the flexible PI substrate has been implemented and demonstrated for the acquisition of bio‐potential signals. The AFE system consists of three monolithically integrated constituent components: a bias‐filter circuit, a 4‐stage differential amplifier, and an additional notch filter. Listed in **Figure** **9** are comparisons of the four metrics of *A*_d_, *CMRR*, *GBWP*, and *η* of the state‐of‐the‐art AFE systems implemented using a variety of TFT technologies. Even with the incorporation of an additional notch filter, the present AFE system still exhibits a record‐setting *η* of 86 kHz mm^−2^. Requiring no supplementary off‐substrate signal‐conditioning components and occupying an area ≈11 mm^2^, the stand‐alone AFE system has been applied to both electromyography and electrocardiography.

## 6 Experimental Section

### Fabrication of the AFE System

The AFE system and its constituent TFTs and capacitors were constructed on 10‐µm‐thick PI coated on a glass carrier substrate. A stacked buffer layer consisting of a 300‐nm‐thick silicon oxide (SiO_x_) on a 200‐nm‐thick silicon nitride (SiN_y_) was deposited on the PI in a plasma‐enhanced chemical vapor deposition (PECVD) equipment at 300 °C using silane, nitrous oxide, and ammonia as the source gases. A 150‐nm‐thick molybdenum (Mo) acting as the BG electrodes of the TFTs and the first layer of interconnection for the AFE system was sputtered on the buffer layer and patterned in mixed phosphoric, nitric, and acetic acids at room temperature. A 50‐nm‐thick PECVD SiN_y_ on a 75‐nm‐thick PECVD SiO_x_ stacked dielectric was next deposited at 300 °C. A 20‐nm‐thick amorphous IGZO active layer was sputtered at room temperature in a mixed O_2_/argon atmosphere using a target with a molar ratio of In_2_O_3_:Ga_2_O_3_:ZnO = 1:1:1 at a total pressure of 3 mTorr. Some of the samples were next treated by direct exposure to a capacitively coupled CF_4_ plasma for 10 min in PECVD equipment at 300 °C with a power of 30 W, a pressure of 550 mTorr, and a CF_4_ flow rate of 400 sccm, thus forming IGZO: F. The TFT active islands were subsequently patterned in 1/2000 hydrofluoric acid solution and capped with a 300‐nm‐thick gas‐permeable PECVD SiO_x_ passivation layer. Contact holes were opened in an inductively coupled plasma etcher running a sulfur hexafluoride chemistry. This was followed by the formation of the S/D stacked electrodes and a second layer of circuit interconnection consisting of 300‐nm‐thick aluminum (Al) on 50‐nm‐thick Mo. Wet etching using the same mixed acid was carried out to pattern the stacked Al on Mo. The TFTs were subsequently annealed at 300 °C in O_2_ for 4 h. Last, the PI together with circuits including the AFE systems was exfoliated from the carrier substrate using a laser‐liftoff process.

### Device Modeling, Circuit Simulation, and Layout Design

The device modeling, circuit simulation, and layout design were carried out using the Empyrean AetherFPD. Level 61 PRI, amorphous silicon‐based TFT model card was chosen to model the IGZO and IGZO: F TFTs.

### Electrical Characterizations of the TFTs, Capacitors, and AFE System

The TFT, capacitor, and AFE measurements were carried out in a regular laboratory environment. TFTs were characterized using an Agilent 4156C Semiconductor Parameter Analyzer. Capacitors were characterized using a Keysight E4980A LCR Meter. During the measurement of the amplifier and the notch filter, the input signals were generated using a FeelTech FY200S Dual Channel Arbitrary Function Signal Generator, and the output signals were rendered on a Tektronix TDS 2012C Oscilloscope. An Agilent 4156C was used as the power supply for the acquisition of the EMG and ECG signals. Three commercial gel electrodes were used and directly connected to the inputs of the AFE system, and the output of the AEF system was directly connected to the Tektronix Oscilloscope. The participant in the experiment shown in Figure 8 is author Runxiao Shi, who has given his consent to publish the data. The AFE testing on the skin surface does not require ethical committee approval because the experiments are only on the surface of the human body, are not invasive, and do not affect the health of the person physically or psychologically. The only participant in the experiment was Runxiao Shi, and no identifiable private signals were collected.

## Conflict of Interest

The authors declare no conflict of interest.

## Supporting information

Supporting Information

## Data Availability

The data that support the findings of this study are available on request from the corresponding author. The data are not publicly available due to privacy or ethical restrictions.
