# Supplementary material for: An Integrated Analog Front‐End System on Flexible Substrate for the Acquisition of Bio‐Potential Signals
Source: Adv Sci (Weinh). 2023 Mar 3;10(13):2207683. doi: 10.1002/advs.202207683 (PMC10161121; doi:10.1002/advs.202207683)
Supplement: Supplementary file 1 — Supporting Information [file ADVS-10-2207683-s001.pdf]

ADVANCED SCIENCE
Open Access

Supporting Information

for *Adv. Sci.*, DOI 10.1002/advs.202207683

An Integrated Analog Front-End System on Flexible Substrate for the Acquisition of Bio-Potential Signals

Runxiao Shi, Xuchi Liu, Tengteng Lei, Lei Lu, Zhihe Xia and Man Wong**

An Integrated Analog Front-End System on Flexible Substrate for the Acquisition of Bio-Potential Signals

Runxiao Shi[1], Xuchi Liu[1], Tengteng Lei[1], Lei Lu[2], Zhihe Xia[1], and Man Wong[1]

[1]State Key Laboratory of Advanced Displays and Optoelectronics and Technologies, Department of Electronic and Computer Engineering, The Hong Kong University of Science and Technology, Hong Kong, China

[2]School of Electronic and Computer Engineering, Peking University, Shenzhen, China

Supporting Information

Table S1. Amplitude and bandwidth of bio-potential signals.

Bio-Potential	Amplitude (mV)	Effective Frequency (Hz)
EMG	0.1-1	1-500
ECG	0.1-0.5	1-150
EOG	0.05-0.1	0.1-50
EEG	0.001-0.1	0.5-50

Table S2. Half-cell potential of typical electrode-electrolyte pairs.

Metal	Reaction	Half-cell potential (V)
Gold	$Au^{+} + e^{-} = Au$	+1.692
Silver	$Ag^{+} + e^{-} = Ag$	+0.7996
Silver	$Ag + Cl^{-} = AgCl + e^{-}$	+0.223
Copper	$Au^{+} + e^{-} = Au$	+0.342
Lead	$Pb^{2+} + 2e^{-} = Pb$	–0.123
Nickel	$Ni^{2+} + 2e^{-} = Ni$	–0.257
Cadmium	$Cd^{2+} + 2e^{-} = Cd$	–0.403
Iron	$Fe^{2+} + 2e^{-} = Fe$	–0.447
Iron	$Fe^{3+} + 3e^{-} = Fe$	–0.037
Zinc	$Zn^{2+} + 2e^{-} = Zn$	–0.762
Aluminum	$Al^{3+} + 3e^{-} = Al$	–1.662

Table S3. Performance of the proposed AFE system compared to published state-of-the-art systems.

Ref.	Moy T [18]	Fuketa H [26]	Sugiyama M [19]	Xu Y [40]	Garripoli C [17]	Zulqarnain M [23]	Zulqarnain M [22]	This work
TFT Process	a-Si	organic	organic	ITZO	IGZO	IGZO	IGZO	IGZO
Area (mm^2)	~ 90	~ 126	~ 170	41.0	11.2	37.0	5.4	11.1
A_d (dB)	~ 20	~ 29	~ 38	20	24	22.8	22	59.6
CMRR (dB)	~ 50	-	~ 52	42	-	-	-	75.7 dB
Bandwidth (Hz)	~ 400	~ 100	~ 100	5 k	5.3 k	3.3 k	3 kHz	1 kHz
GBWP (Hz)	~ 4 k	~ 2.8 k	~ 5.6 k	50 k	84 k	45.6 k	37.8 k	955 k
GBWP/Area (Hz/mm^2)	~ 44.4	~ 22.3	~ 46.7	1.2 k	7.5 k	1.2 k	7 k	86 k
Off-substrate Components	Off-substrate capacitor	No	No	No	No	Off-substrate capacitor	Off-substrate capacitor	No
In-vitro measurement	EEG	EMG	ECG	ECG	EMG	ECG	ECG	ECG/EMG

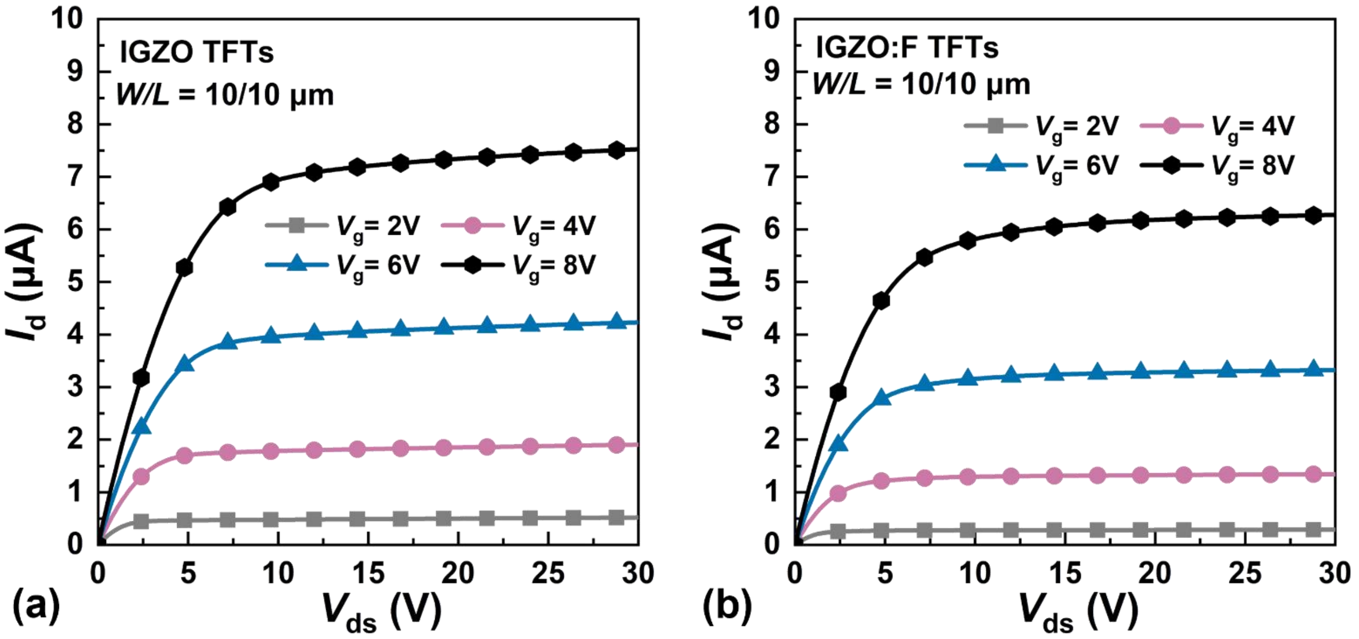

Fig. S1 | The output characteristics of a, IGZO and **b,** IGZO:F TFTs.

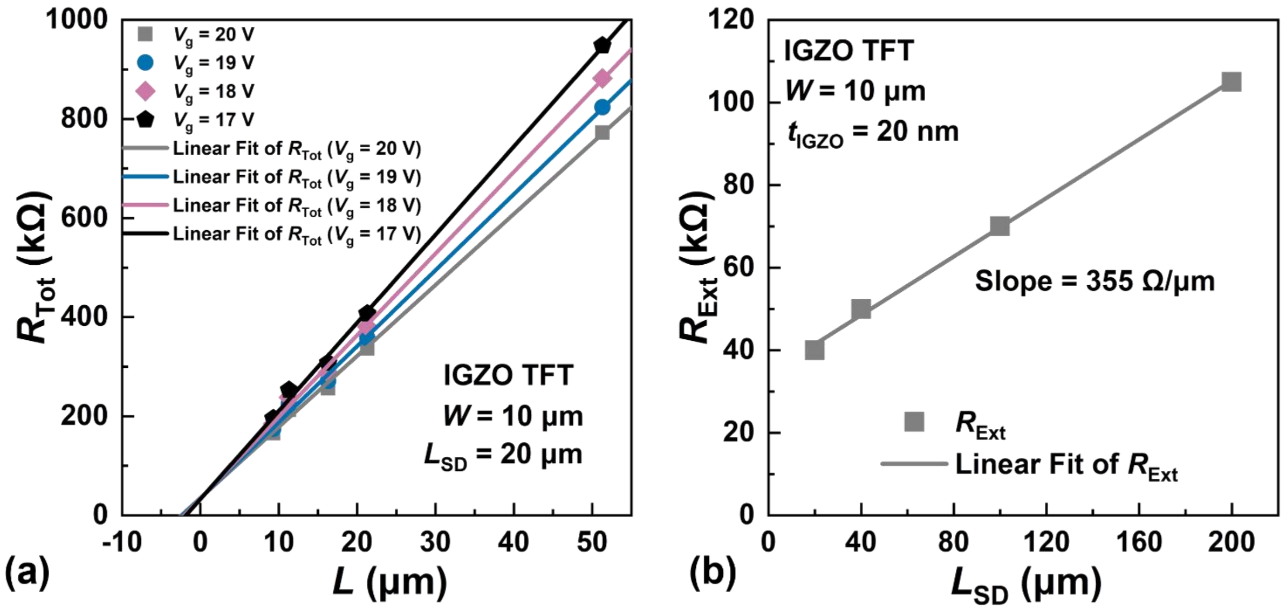

Fig. S2 | The extraction of the resistivity of conductive IGZO. a, R_{Tot} of the IGZO TFTs with L_{SD}= 20 μm and W= 10 μm at different V_{g}. **b,** The dependence of R_{Ext} on L_{SD} of IGZO TFTs with W=10 μm.

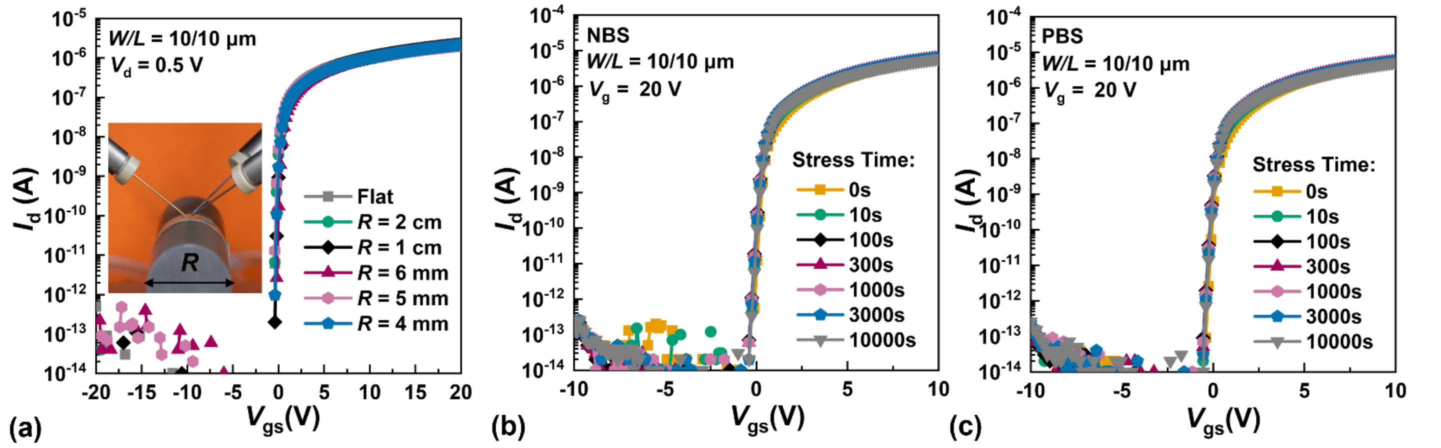

Fig. S3 | Characteristics of the IGZO:F TFT under mechanical and electrical stress. **a,** Under mechanical stress along the *W* of channel. **b,** Under negative-bias stress (NBS) and **c,** Under positive-bias stress (PBS).

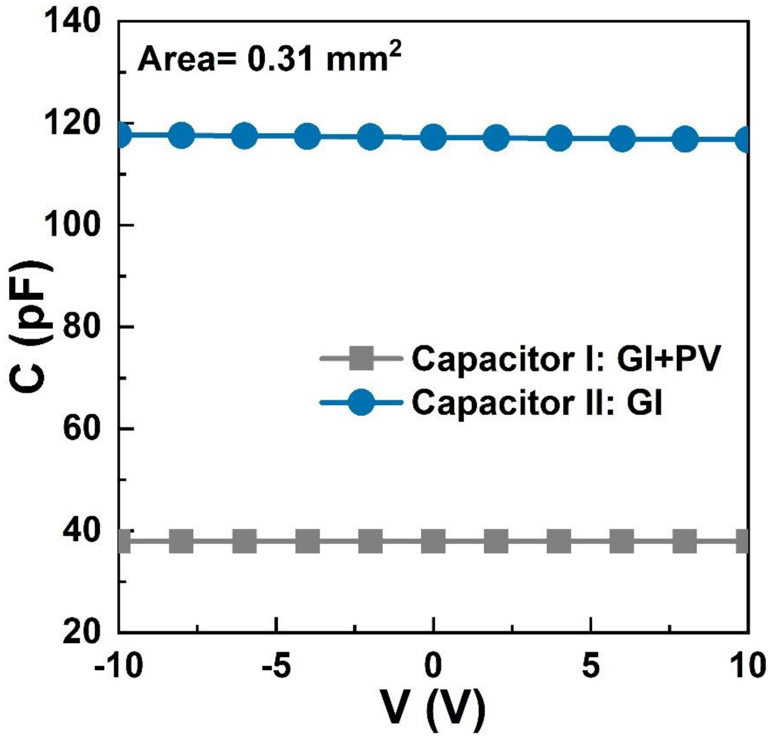

Fig. S4 | Capacitance-voltage characteristics of Capacitors I and II. The relatively constant capacitance across the bias range of −10 to 10 V reflects a negligible depletion of the charge carriers in the top conductive IGZO plate of Capacitor II.

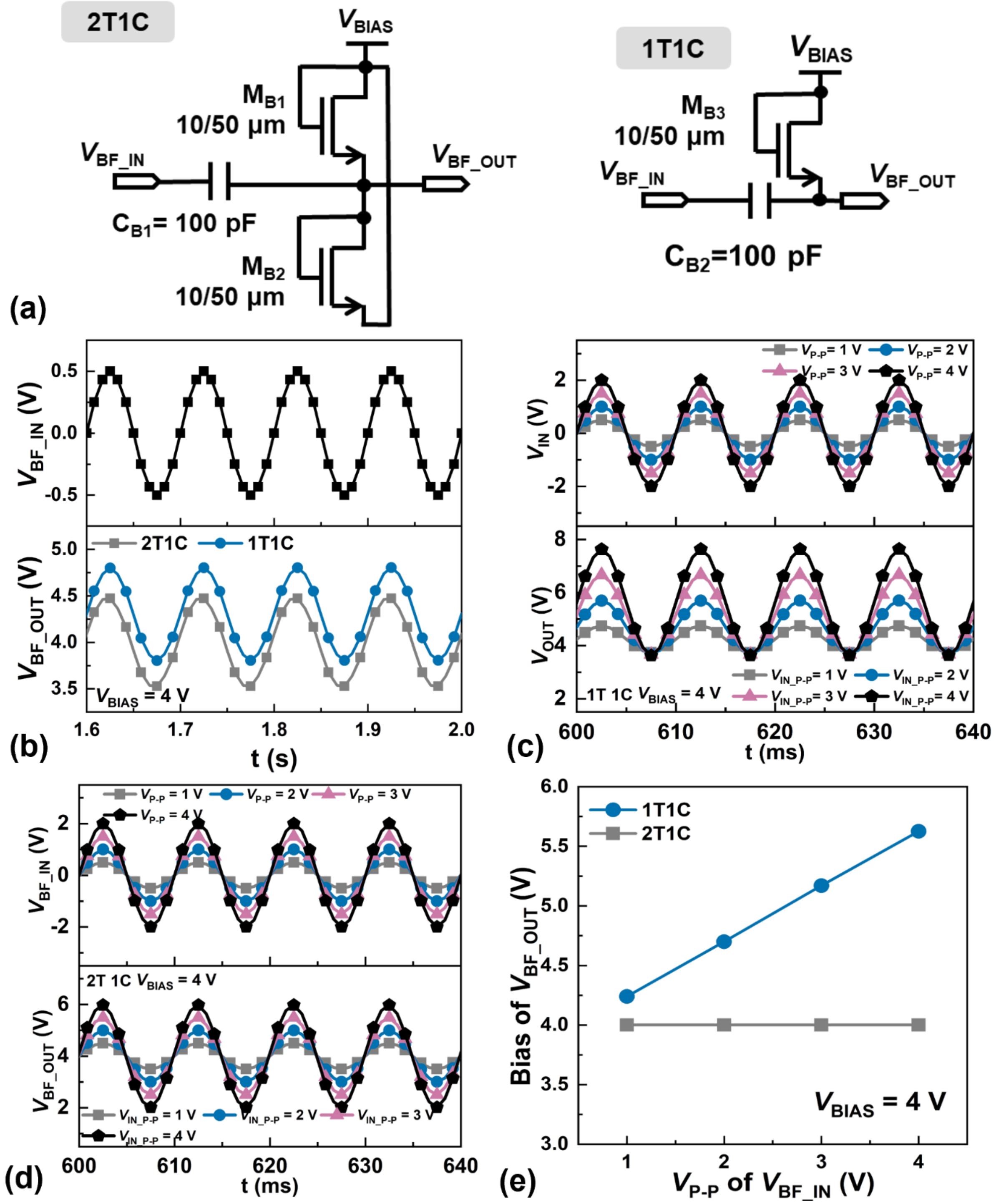

Fig. S5 | The role of the discharging TFT M_{B2} in the 2T1C bias-filter circuit. a, Circuit diagrams of the 2T1C and 1T1C designs. **b,** Simulation results for designs subjected to a 10-Hz sinusoidal input waveform with $V_{\mathrm{P-P}} = 1$ V. The DC component of the $V_{\mathrm{BF\_OUT}}$ of the 2T1C is accurately sets at $V_{\mathrm{BIAS}} = 4$ V, while that of the 1T1C design exhibits a deviation from V_{BIAS}. **c, d,** The respective performance of 1T1C and 2T1C bias-filter for input waveform with different $V_{\mathrm{P-P}}$. **e,** The DC component of the $V_{\mathrm{BF\_OUT}}$ of the 1T1C design shows a dependence on the $V_{\mathrm{P-P}}$ of the $V_{\mathrm{BF\_IN}}$. The deviations observed in the 1T1C design are caused by the lack of a discharging path for the capacitor.

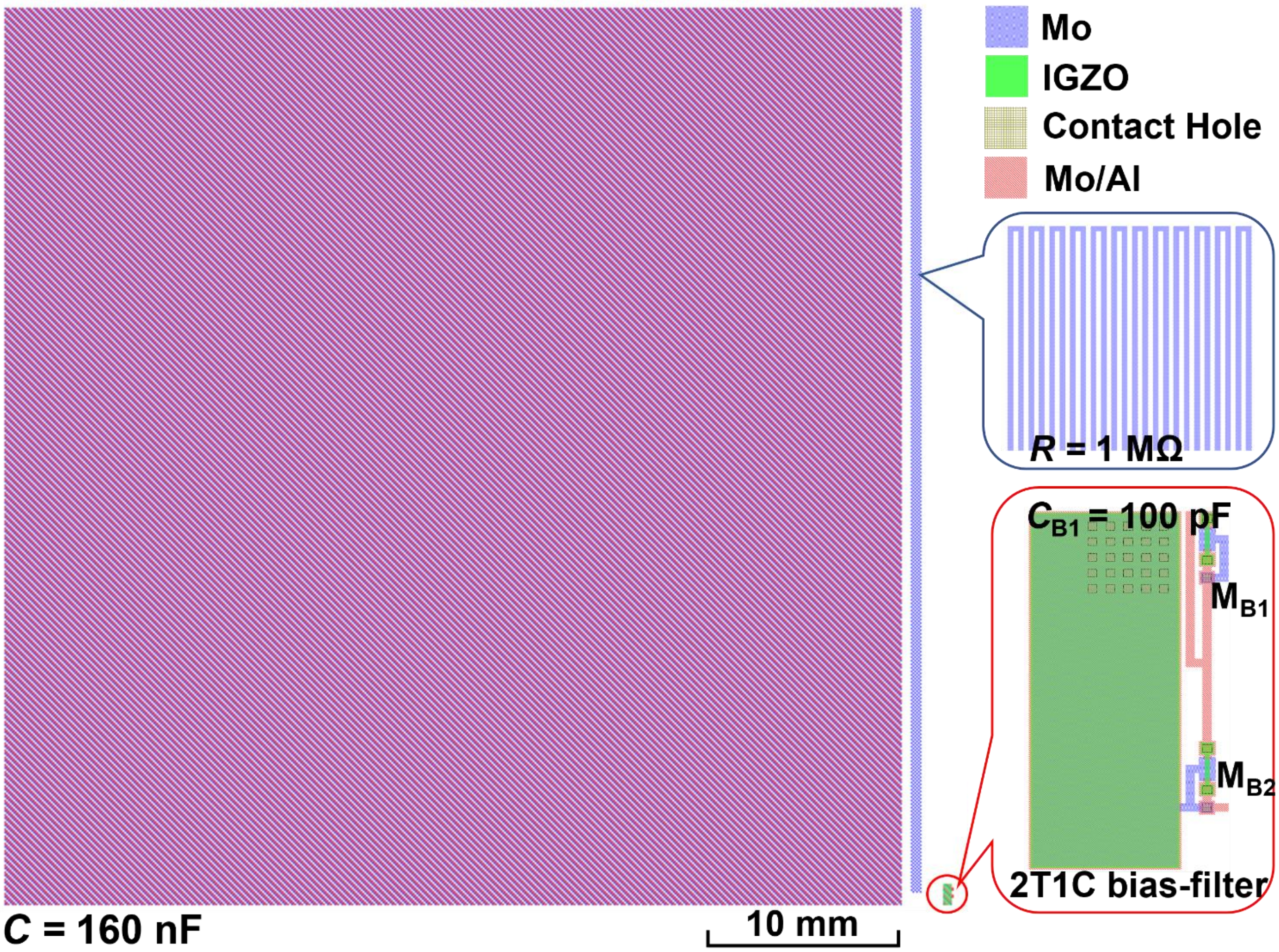

Fig. S6 | Comparison of the layouts of the 1R1C and the 2T1C bias-filter designs. The footprint of the latter is more than 1600 times smaller than that of the former.

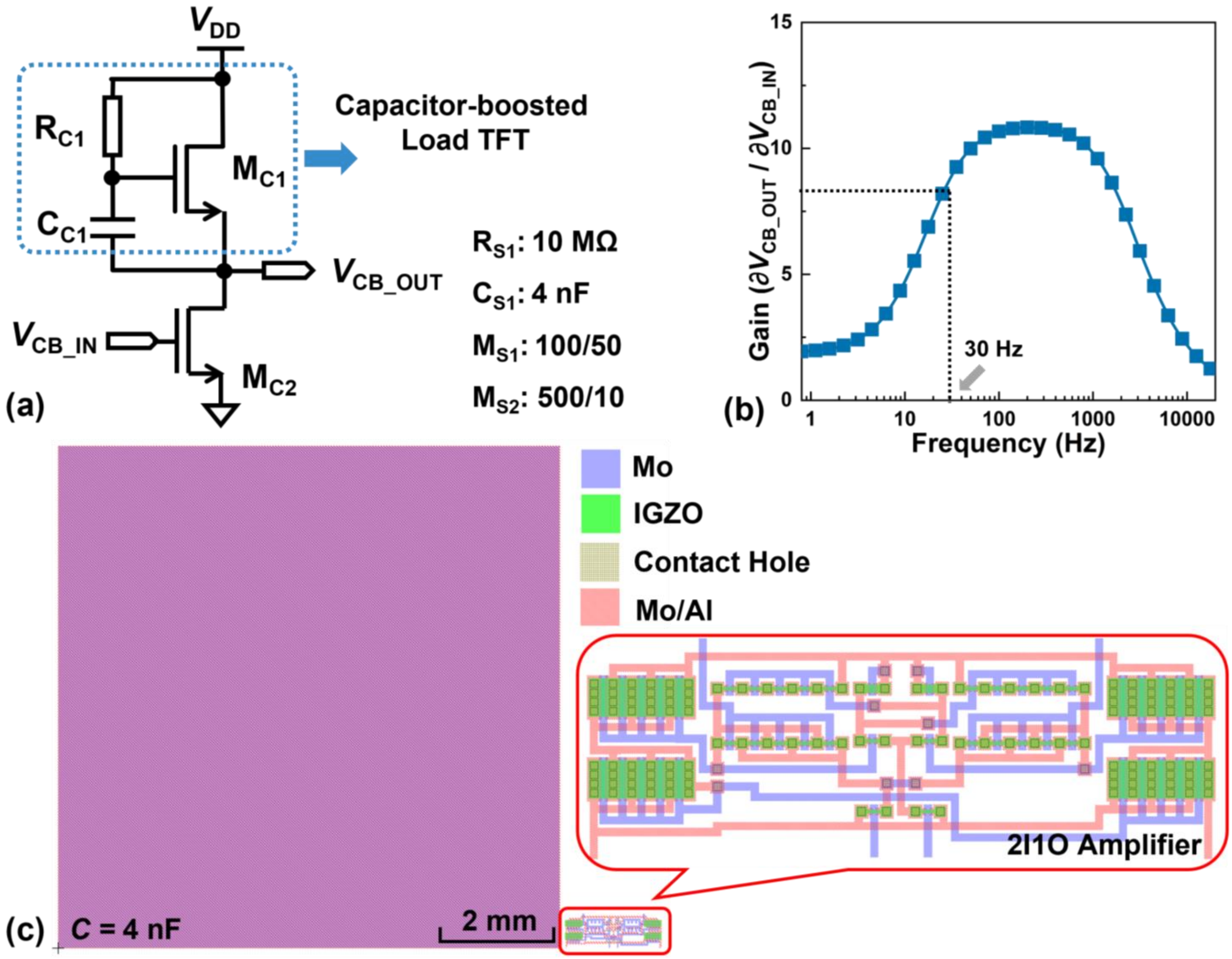

Fig. S7 | Capacitor-boosting method to increase the A_d of an amplifier. (a) Capacitor-boosted load TFT to shunt the gate and source terminals of the load TFT, thus eliminating the shunting transconductance and increasing A_d. C_{S1} must be relatively large for the gate-source shunting to be effective even at the relatively low ~1 Hz of bio-potential signals. (b) Although a large C_{S1} of 4 nF is used in the capacitor-boosted load TFT, the f_C of ~30 Hz the amplifier is still relatively large. This will cause a loss of the low-frequency portion of the bio-potential signal. (c) Layout comparison of a 4-nF capacitor and a 2I2O amplifier used in the present AFE system, demonstrating that capacitor-boosting does not have an advantage in reducing S.

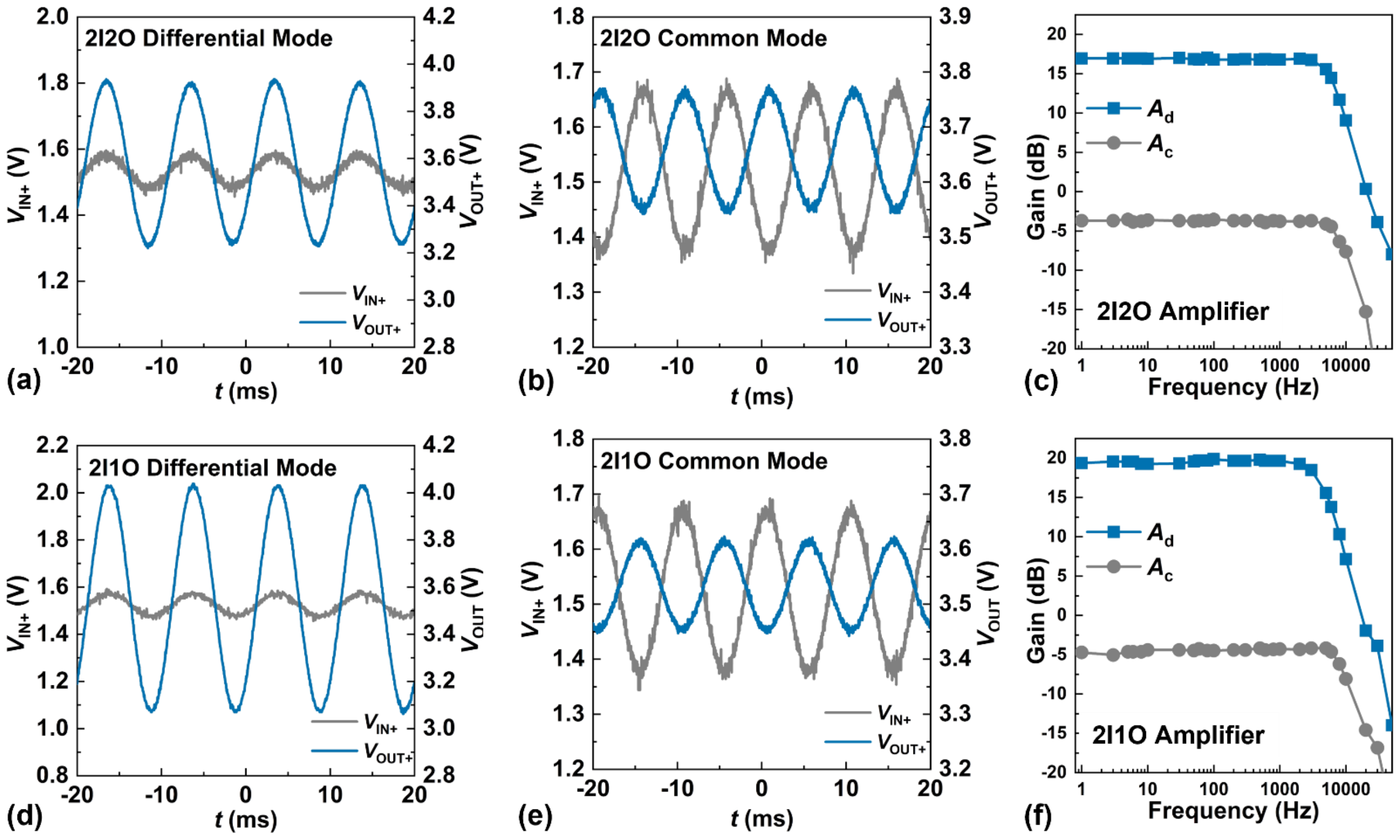

Fig. S8 | The performance of single differential amplifier stages characterized individually. For a 2I2O amplifier: **a,** Measured input and output waveforms with a 10-Hz sinusoid differential-mode input with $V_{\mathrm{P-P}} = 100$ mV. **b,** Measured input and output waveforms with 10-Hz sinusoid common-mode input with $V_{\mathrm{P-P}} = 300$ mV. **c,** Frequency response characteristic, showing the $A_{\mathrm{d}} \approx 17.0$ dB and $A_{\mathrm{c}} \approx -3.7$ dB. For a 2I1O amplifier: **d,** Measured input and output waveforms with 10-Hz sinusoid differential-mode input with $V_{\mathrm{P-P}} = 100$ mV. **b,** Measured input and output waveforms with 10-Hz sinusoid common-mode input with $V_{\mathrm{P-P}} = 300$ mV. **c,** Frequency response characteristics, showing the $A_{\mathrm{d}} \approx 19.4$ dB and $A_{\mathrm{c}} \approx -4.7$ dB. For a 4-stage cascade, a maximum $A_{\mathrm{d}} \approx 70.4$ dB can be estimated.

Table S4. The values of the bias settings for obtaining the measurement results of Fig. 6c.

f_{CEN}	**40 Hz**	**50 Hz**	**60 Hz**
V_{DD}	15 V	15 V	15 V
V_{SF}	1 V	1 V	1 V
V_{R1}	7.7 V	8.1 V	8. 5 V
V_{R2}	6. 4 V	6.9 V	7. 4 V
**DC of** V_{NF_IN}	5 V	5 V	5 V
V_{FB}	1 V	1 V	1 V

Table S5. The values of the bias settings for obtaining the measurement results of Fig. 6d.

V_{FB}	**1 V**	**5 V**	**7 V**
V_{DD}	15 V	15 V	15 V
V_{SF}	1 V	1 V	1 V
V_{R1}	8.1 V	8. 1 V	8. 1 V
V_{R2}	6.9 V	7.4 V	7.9 V
**DC of** V_{NF_IN}	5 V	5 V	5 V
**Bandwidth**	390 Hz	280 Hz	180 Hz
